# The relationship between dietary magnesium intake, stress level and headache in academic and administrative staff

**DOI:** 10.3389/fnut.2026.1694363

**Published:** 2026-01-26

**Authors:** Naciye Kılıç, Nihal Zekiye Erdem

**Affiliations:** 1 Department of Nutrition and Dietetics, Institute of Health Sciences, Istanbul Medipol University, Istanbul, Türkiye; 2 Department of Nutrition and Dietetics, College of Health Sciences, Istanbul Medipol University, Istanbul, Türkiye

**Keywords:** academic and administrative staff, dietary magnesium, headache, nutrition, stress

## Abstract

**Background:**

Magnesium is an important mineral that plays a role in many biochemical reactions in the body. Since it plays a role in many mechanisms of the body, it is thought that it may also have effects on stress and headaches.

**Methods:**

The study was conducted with the participation of a total of 150 volunteer academic and administrative staff aged between 19 and 65. Participants were evaluated through general information, anthropometric measurements, Headache Impact Test-6 (HIT-6) and Perceived Stress Level Scale-10 (PSS-10), 3-day food consumption record and frequency of consumption of magnesium-rich foods questionnaire.

**Results:**

The mean age of the participants was 31 ± 6.283 years for men and 28.55 ± 5.294 years for women. The Headache Impact Test-6 score was higher in the group with inadequate magnesium intake (*p* < 0.05). Although the Perceived Stress Level Scale-10 score was also higher in the group with inadequate magnesium intake, the result was not significant (*p* > 0.05). A negative correlation was found between dietary magnesium and HIT-6 and PSS-10 scores (*r* = −0.183, *r* = −0.197, respectively; *p* < 0.05). A positive correlation was also found between the scales (*r* = 0.456; *p* < 0.01).

**Conclusion:**

These findings suggest that lower dietary magnesium intake is associated with higher headache impact and perceived stress in academic and administrative staff.

## 1 Introduction

Magnesium (Mg) is an essential mineral, the 4th most abundant cation in the body and a cofactor in more than 300 biochemical reactions. It is involved in many reactions such as inhibition of neuronal excitation and vasospasm, reduction of the formation of inflammatory substances, mitochondrial oxidative phosphorylation, improvement of serotonin receptor transmission (1, 2).

Stress, which usually occurs as a response to external stressors, has become a common problem of modern life (3). Among the external factors that play a role in the emergence of stress, the effect of nutritional style on stress has a significant value (4). In terms of nutrients, a strong relationship between Mg and stress was first recognized in the 1990s. Galland and Seelig (5), revealed that Mg and stress have a bidirectional relationship, that there is a decrease in serum Mg in case of stress, and that stress level increases in Mg deficiency.

Depending on the type and duration of exposure to stress, it can lead to short-term effects (increased blood pressure, respiratory rate, wakefulness) or long-term effects (cognitive and memory impairment) (6, 7). In addition, another health problem that stress can cause is headaches (8).

Headaches are one of the most common ailments in human history (9). According to the International Classification of Headache Disorders-3 (ICHD-3) criteria, headaches are divided into two groups: primary and secondary. The primary group includes headaches that occur without being related to the pathology of any disease developing in the body. The secondary group includes headaches that occur due to disease or pathology. Migraine, tension-type headache (TTH) and trigeminal autonomic cephalalgia (cluster headache), which are linked to the primary group and are the most common headaches worldwide (10). There are many risk factors for headaches. These include lifestyle-related stress and diet-related Mg deficiency (11, 12). A study conducted in Türkiye identified stress as the most common trigger of migraine (79% of the sample) (13). In studies conducted with magnetic resonance spectroscopy, it was observed that Mg levels were decreased in the serum, saliva and cerebrospinal fluid of migraine patients, and the decrease was evident during and between migraine attacks (14, 15). In patients with TTH, ionized Mg levels in serum, salivary secretions and platelets decreased (16, 17). It has also been reported that computer use for more than 12 h a day, shift work for more than 6 nights in a month and obesity are among the risk factors for TTH (18, 19).

Therefore, the aim of this study was to examine the effect of dietary Mg intake on stress and headache as well as the relationship between stress and headache in academics and administrative staff.

## 2 Methods

The data of the study were obtained through a questionnaire. Voluntary consent was obtained from the participants with an informative letter at the beginning of the study.

### 2.1 Study design and study population

The sample size of the study was calculated using the G*Power statistical analysis program and it was determined that at least 146 participants were required for the study with 99% power and 5% margin of error. This is a cross-sectional study conducted with 150 academic and administrative staff between November 2023 and January 2024. A total of 150 individuals, 58 males and 92 females, between the ages of 19–65, working at universities in Istanbul participated.

### 2.2 Exclusion criteria

Individuals taking Mg supplements, taking regular medication, diagnosed with neurological disease, metabolic syndrome, renal or hepatic insufficiency, diagnosed with cancer, thyroid disease, diagnosed with polycystic ovary syndrome, experiencing headache belonging to the secondary group according to ICHD-3-beta classification, diagnosed with psychiatric disease, unable to make decisions on their own, under 19 and over 65 years of age, with a body mass index (BMI) above 25 kg/m^2^ and below 18.5 kg/m^2^, pregnant and breastfeeding individuals were not included in the study.

### 2.3 Data collection and evaluation

In the first part of the questionnaire, sociodemographic characteristics (gender, age, educational status and occupation), anthropometric measurements (body weight, height and BMI), smoking, alcohol use and vitamin-mineral intake were questioned. Headache Impact Test-6 (HIT-6) was used to assess headache severity in individuals. Perceived Stress Scale-10 (PSS-10) was used to asses the stress level of individuals. In the last part of the questionnaire, a 3-day food consumption record and magnesium-rich food consumption frequency form were used to determine dietary habits and dietary Mg intake.

### 2.4 Anthropometric measures

Body weight (kg) and height (cm) of all individuals were measured by the researcher. Body mass index (kg/m^2^) values were also calculated. Based on the classification of the World Health Organization, the participants were classified as normal weight (20).

### 2.5 Headache Impact Test (HIT-6)

The Headache Impact Test is a short questionnaire designed by Bayliss et al. (21) to describe how individuals feel in their daily lives and how often they are unable to perform certain activities (work, school, housework, social activities) due to headaches. It is reported in the literature that HIT-6 facilitates the evaluation and follow-up of individuals with headache. The validity and reliability study of the scale in Türkiye was conducted by Dikmen et al. (22) between 2017 and 2018. There are 6 questions in this test and the questions are scored according to the answers given. With the HIT-6 test, which aims to measure the degree of impact of headaches, individuals were divided into four groups. The severity of impact category is as follows: headache with little or no impact (49 points or less), headache with some impact (50–55 points), headache with substantial impact (56–59 points) and headache with severe impact (60–78 points).

### 2.6 Perceived Stress Scale (PSS-10)

The perceived stress scale test was developed by Cohen et al. (23) in 1983 to measure the levels of stress affecting the social life of individuals. The validity and reliability study of the scale in Türkiye was conducted by Eskin et al. (24) in 2013. There are 10 questions in the PSS-10. The total score given to the questions varies between 0 and 40. The stress category is evaluated as low stress (0–13 points), moderate stress (14–26 points), and high perceived stress (27–40 points).

### 2.7 Evaluation of dietary consumption and magnesium intake

Total energy and nutrient intake of individuals was obtained with a 3-day retrospective food consumption record form. In order to evaluate the amounts of consumed foods without error, the book “Food and Nutrition Photograph Catalogue: Measurements and Quantities” was used (25). The Computer-Assisted Nutrition Program, Nutrition Information System (26) was used to calculate the energy and nutrient intakes provided by consumed foods. In order to better understand dietary Mg intake, individuals were also administered a Mg-rich food consumption frequency survey. The Turkish Specific Nutrition Guide (27) emphasizes that the daily adequate Mg intake value for men aged 19–65 is 350 mg and for women, it is 300 mg. Accordingly, for women, <300 mg/day is considered inadequate Mg intake, ≥300 mg/day is considered adequate Mg intake; for men, <350 mg/day is considered inadequate Mg intake, ≥350 mg/day is considered adequate Mg intake.

### 2.8 Statistical analyses

Statistical analysis of the data was performed using SPSS (IBM, Chicago, IL, USA) 26.0 package program. In addition to descriptive statistical methods (Mean, Standard Deviation, Median, Frequency, Percentage, Minimum, Maximum), the suitability of the data for normal distribution was evaluated with the Kolmogorov–Smirnov Test. Mann Whitney *U* test was used for group comparisons of quantitative (numerical) data that did not show normal distribution, and independent sample *t-*test was used for group comparisons that showed normal distribution. In order to determine the relationship between qualitative (categorical) data, Chi square test was used when the expected frequency of cells was high (more than 5) and Fisher exact test was used when it was low (less than 5). In order to determine the relationship between quantitative data, Pearson test was used in case of normal distribution and Spearman test was used in case of non-normal distribution. Significance was evaluated at *p* < 0.05.

## 3 Results

### 3.1 Characteristics of the study population

The study consisted of a total of 150 individuals, 58 men (38.7%) and 92 women (61.3%). The mean age of the men participating in the study was 31 ± 6.283 years, while the mean age of the women was 28.55 ± 5.294 years. The Chi-square test was used to examine whether there was a significant relationship between the education and occupation variables and the gender variable, and no significant relationship was found with the gender variable in terms of both education status and occupation (*p* > 0.05) (Table 1). When grouped according to gender according to occupational status, 43.2% of academicians were male and 56.8% were female. Among administrative personnel, 34.2% were male and 65.8% were female. 49.3% of academicians and 50.7% of administrative personnel participated in the study. As a result of the examination, no significant relationship was observed between occupation and gender variables (*p* > 0.05).

**Table 1:** Characteristics of the study population.

Characteristic	Men		Women		*p*-values
	%	*n*	%	*n*	
	38.7	58	61.3	92	
Age					
Mean X ± S	31 ± 6.283		28.55 ± 5.294		
Education *n* (%)					
Bachelor’s	24 (36.4)		42 (63.6)		0.796
Master’s	22 (42.3)		30 (57.7)		
Doctorate	12 (37.5)		20 (62.5)		
Occupation *n* (%)					
Academic staff	32 (43.2)		42 (56.8)		0.256
Administrative staff	26 (34.2)		50 (65.8)		

In terms of smoking status, 11.4% (4 individuals) of smokers received adequate Mg, while 88.6% (31 individuals) received inadequate Mg. There was no significant relationship between smoking status and dietary Mg intake (*p* > 0.05). In terms of alcohol consumption, 9.5% (4 people) of the individuals who consumed alcohol received adequate Mg, while 90.5% (38 people) received inadequate Mg. Of the individuals who took adequate Mg and consumed alcohol, 75% were male and 25% were female. Similarly, 55.3% of the individuals who took inadequate Mg and consumed alcohol were male and 44.7% were female. According to the analysis, no significant relationship was found between alcohol consumption and Mg intake (*p* > 0.05). Among the individuals taking vitamin-mineral supplements, 5.4% (2 individuals) received adequate Mg, while 94.6% (35 individuals) received inadequate Mg. Of the individuals who received adequate Mg and vitamin-mineral supplements, 69.2% were male and 30.8% were female. Similarly, 40% of the individuals taking inadequate Mg and vitamin-mineral supplements were male and 60% were female. According to the analysis, no significant relationship was found between vitamin-mineral intake status and Mg intake (*p* > 0.05). The findings obtained by analyzing whether there was a difference in terms of body weight, height and BMI values of the individuals and their dietary Mg intake status were given. Accordingly, a statistically significant difference was found in the body weight and height variables in terms of Mg intake status (*p* < 0.05), while no significant difference was found in the BMI variable (*p* > 0.05) (Table 2). The mean and standard deviation values of the energy and nutrients of the individuals according to the group receiving adequate and inadequate Mg are given. Accordingly, a statistically significant difference was found in energy, carbohydrate, protein, fat, pulp, saturated fatty acid (SFA), monounsaturated fatty acid (MUFA), polyunsaturated fatty acid (PUFA), cholesterol, vitamin E, B1, B2, B12, niacin, folate, sodium (Na), potassium (K), calcium (Ca), Mg, phosphorus (P), iron, zinc, omega-3 intake in terms of Mg intake (*p* < 0.05). No statistically significant difference was found in carbohydrate, protein and fat percentage, alcohol, alcohol percentage, vitamin A, vitamin K, vitamin C intake in terms of Mg intake variable (*p* > 0.05) (Table 3).

**Table 2:** Characteristics of study population and anthropometric measurements.

Characteristic and anthropometric measurements	Adequate Mg intake			Inadequate Mg intake			
	Men	Women	Total	Men	Women	Total	*p*-values
Smoking, *n* (%)							
Yes	2 (50)	2 (50)	4	17 (54.8)	14 (45.2)	31	0.104
No	9 (81.8)	2 (18.2)	11	30 (28.8)	74 (71.2)	104	
Alcohol, *n* (%)							
No	8 (72.7)	3 (27.3)	11	26 (26.8)	71 (73.2)	97	0.904
Yes	3 (75)	1 (25)	4	21 (55.3)	17 (44.7)	38	
Vitamin mineral supplements, *n* (%)							
Yes	2 (100)	0 (0)	2	7 (20)	28 (80)	35	0.283
No	9 (69.2)	4 (30.8)	13	40 (40)	60 (60)	100	
Weight (kg)							
Mean X ± S	77.36 ± 9,179	61.5 ± 3.873	73.13 ± 10.776	75.13 ± 7.146	58.34 ± 5.843	64.19 ± 10.204	0.004
Height (cm)							
Mean X ± S	181.64 ± 6.607	166 ± 5.715	177.47 ± 9.456	178.49 ± 4.731	164.31 ± 5.865	169.24 ± 8.718	0.003
BMI (kg/m^2^)							
Mean X ± S	23.39 ± 1.809	22.43 ± 2.659	23.13 ± 2.012	23.55 ± 1.544	21.62 ± 2.018	22.29 ± 2.079	0.099

Mg: Magnesium; BMI: body mass index.

**Table 3:** Participants’ energy, macro- and micronutrient intakes.

Macro- and micronutrients	Adequate Mg intake			Inadequate Mg intake			***p*-values**
	Men	Women	Total	Men	Women	Total	
	X ± S	X ± S	X ± S	X ± S	X ± S	X ± S	
Energy (kkal)	2283.67 ± 399.132	1559.32 ± 484.687	2090.51 ± 523.511	1624.81 ± 366.149	1350.8 ± 305.996	1446.2 ± 352.108	<0.001
Carbohydrate (g)	226.65 ± 54.694	162.79 ± 55.177	209.62 ± 60.362	169.63 ± 45.784	139.78 ± 37.113	150.17 ± 42.632	<0.001
Carbohydrate (%)	40.45 ± 5.973	42.25 ± 3.775	40.93 ± 5.405	42.47 ± 4.539	42.28 ± 5.721	42.35 ± 5.323	0.309
Protein (g)	99.99 ± 24.759	61.75 ± 11.892	89.79 ± 27.829	69.63 ± 18.018	56.77 ± 15.213	61.25 ± 17.308	<0.001
Protein %	17.91 ± 3.91	17 ± 4.967	17.67 ± 4.047	17.64 ± 3.151	17.26 ± 3.402	17.39 ± 3.31	0.967
Fat (g)	105.83 ± 25.92	72.53 ± 27.822	96.95 ± 29.635	72.24 ± 16.83	61.39 ± 16.058	65.17 ± 17.076	<0.001
Fat (%)	41.18 ± 6.306	40.75 ± 3.304	41.07 ± 5.548	39.68 ± 3.701	40.36 ± 4.661	40.13 ± 4.349	0.484
Fiber (g)	25.08 ± 6.787	15.26 ± 5.628	22.46 ± 7.74	16.77 ± 5.007	14.84 ± 5.47	15.51 ± 5.375	0.001
Alcohol (g)	0.98 ± 3.03	0.02 ± 0.045	0.73 ± 2.599	0.75 ± 3.332	0.08 ± 0.161	0.32 ± 1.982	0.952
Alcohol (%)	0.36 ± 1.206	0 ± 0	0.27 ± 1.033	0.23 ± 1.127	0 ± 0	0.08 ± 0.67	0.183
SFA	38.42 ± 10.159	28.19 ± 6.254	35.69 ± 10.201	28.06 ± 7.669	24.28 ± 7.32	25.6 ± 7.632	<0.001
MUFA	38.88 ± 11.029	25.24 ± 10.8	35.24 ± 12.284	25.94 ± 6.575	21.03 ± 5.94	22.74 ± 6.578	<0.001
PUFA	19.15 ± 5.541	12.74 ± 7.932	17.44 ± 6.635	12.35 ± 4.205	10.64 ± 4.58	11.23 ± 4.512	<0.001
Cholesterol (mg)	431.12 ± 221.565	203.43 ± 68.301	370.4 ± 216.626	309.91 ± 152.614	219.88 ± 95.286	251.22 ± 125.472	0.022
A vit (mcg)	1682.53 ± 2877.404	584.88 ± 171.099	1389.82 ± 2484.475	1470.63 ± 2255.175	720.81 ± 342.083	981.86 ± 1396.567	0.641
E vit (mg)	24.28 ± 7.88	12.06 ± 5.651	21.02 ± 9.084	13.87 ± 4.826	12.49 ± 5.399	12.97 ± 5.23	0.001
K vit (mcg)	69.6 ± 41.55	60.45 ± 16.279	67.16 ± 36.159	90.11 ± 81.969	91.72 ± 92.748	91.16 ± 88.837	0.973
B1 (mg)	1.22 ± 0.245	0.68 ± 0.155	1.07 ± 0.332	0.78 ± 0.2	0.67 ± 0.21	0.71 ± 0.212	<0.001
B2 (mg)	1.69 ± 0.544	0.97 ± 0.15	1.5 ± 0.568	1.25 ± 0.474	0.96 ± 0.293	1.06 ± 0.392	0.001
B12 (mcg)	8.32 ± 8.233	3.83 ± 1.502	7.12 ± 7.289	6.53 ± 8.147	3.23 ± 1.55	4.38 ± 5.179	0.003
Niacin (mg)	44.33 ± 16.974	24.99 ± 4.813	39.17 ± 17.003	29.18 ± 8.836	23.74 ± 7.655	25.63 ± 8.463	<0.001
Folate (mcg)	316.03 ± 100.537	251.48 ± 48.106	298.82 ± 92.676	267.93 ± 88.371	213.77 ± 82.825	232.62 ± 88.348	0.008
C vit (mg)	80.76 ± 41.484	70.38 ± 38.101	77.99 ± 39.534	69.73 ± 36.884	76.91 ± 47.176	74.41 ± 43.86	0.623
Sodium (mg)	2754.93 ± 639.049	2492.74 ± 487.548	2685.02 ± 597.53	2714.75 ± 1120.911	1947.36 ± 593.687	2214.52 ± 891.514	0.021
Potassium (mg)	3085.25 ± 382.559	1954.8 ± 490.164	2783.8 ± 650.98	2165.05 ± 548.504	1928.82 ± 551.577	2011.07 ± 559.969	<0.001
Calcium (mg)	927.35 ± 215.623	733.72 ± 162.894	875.72 ± 216.222	767.29 ± 238.06	674.7 ± 217.428	706.93 ± 228.273	0.010
Magnesium (mg)	421.63 ± 58.507	365.51 ± 36.61	406.67 ± 58.244	222.38 ± 56.311	200.41 ± 54.216	208.06 ± 55.743	<0.001
Phosphorus (mg)	1603.92 ± 269.262	962.71 ± 176.302	1432.93 ± 380.255	1098.22 ± 259.147	900.05 ± 243.751	969.04 ± 265.722	<0.001
Iron (mg)	24.05 ± 5.784	13.24 ± 4.668	21.17 ± 7.285	14.23 ± 5.01	14.29 ± 4.285	14.27 ± 4.532	<0.001
Zinc (mg)	88.46 ± 49.91	51.55 ± 25.989	78.62 ± 47.005	43.83 ± 32.839	58.6 ± 27.199	53.46 ± 30.007	0.015
Omega-3 (g)	1.95 ± 0.911	1.59 ± 0.867	1.86 ± 0.884	1.7 ± 1.257	1.21 ± 0.548	1.38 ± 0.89	0.019

SFA: saturated fatty acids; MUFA: monounsaturated fatty acids; PUFA: polyunsaturated fatty acids.

As a result of the examination of the PSS-10 scores of the participants according to gender, it was observed that there was a significant difference between the PSS-10 scores of female and male individuals, and the scores of female individuals (19.85 ± 4.778) were higher than male individuals (15.95 ± 6.929) (*p* < 0.001) (Table 4). When the PSS-10 was analyzed according to gender in terms of classes, it was seen that there was a significant relationship between the “low level stress” and “medium level stress” classes and the gender variable (*p* < 0.05, *p* < 0.001, respectively). It was analyzed whether there was a significant difference in the HIT-6 scores of the participants according to gender and whether there was a significant relationship between the HIT-6 classification and gender. As a result of the analysis, it was observed that there was a significant difference between the HIT-6 scores of female and male individuals, and the scores of female individuals (54.87 ± 7.442) were higher than those of male individuals (48.97 ± 7.31) (*p* < 0.001). The score was examined in terms of classifications, it was seen that there was a significant relationship between the classes of “little or no impact” and “severe impact” and the gender variable (*p* = 0.001, *p* < 0.001, respectively) (Table 4).

**Table 4:** Participants’ PSS-10 and HIT-6 classification.

Determinants	Men	Women	Total	*p*-values
	X ± S	X ± S	X ± S	
PSS-10 score	15.95 ± 6.929	19.85 ± 4.778	18.34 ± 5.995	<0.001
PSS-10 classification				
Low stress, *n* (%)	19 (70.4)	8 (29.6)	27	0.034
Moderate stress, *n* (%)	35 (31.2)	77 (68.8)	112	<0.001
High perceived stress, *n* (%)	4 (36.4)	7 (63.6)	11	0.549
HIT-6 score	48.97 ± 7.31	54.87 ± 7.442	52.59 ± 7.912	<0.001
HIT-6 classification				
Little or no impact, *n* (%)	26 (60.5)	17 (39.5)	43	0.001
Some impact, *n* (%)	22 (40)	33 (60)	55	0.799
Substantial impact, *n* (%)	6 (31.6)	13 (68.4)	19	0.497
Severe impact, *n* (%)	4 (12.1)	29 (87.9)	33	<0.001

PSS: perceived stress scale; HIT: headache impact test.

When the mean values of the scores were examined according to the level of Mg intake and gender, it was observed that both HIT-6 and PSS-10 scores of female individuals were higher than male individuals for both groups with adequate and inadequate Mg intake. As a result of the analysis of Mg intake status for the HIT-6, a significant difference was observed for the two groups (*p* < 0.05). The scores of individuals with inadequate Mg intake were higher than those with adequate Mg intake. However, although there was no significant difference in PSS-10 scores for these two groups (*p* > 0.05), the PSS-10 values of the group with adequate Mg intake were lower than those with inadequate Mg intake (Table 5).

**Table 5:** Participants’ PSS-10 and HIT-6 scores.

	Adequate Mg Intake			Inadequate Mg Intake			*p*-values
	Men	Women	Total	Men	Women	Total	
	X ± S	X ± S	X ± S	X ± S	X ± S	X ± S	
HIT-6	47 ± 8.025	50.5 ± 10.878	47.93 ± 8.598	49.43 ± 7.147	55.07 ± 7.276	53.1 ± 7.693	0.023
PSS-10	13.64 ± 7.061	21.25 ± 2.217	15.67 ± 6.986	16.49 ± 6.862	19.78 ± 4.86	18.64 ± 5.829	0.126

PSS: perceived stress scale; HIT: headache impact test.

Correlations between participants’ dietary Mg intake, age, height, body weight, BMI values, HIT-6 scores, PSS-10 scores and nutrients are given in Table 6. In the table, a positive relationship was found between Mg intake and height, body weight, energy, carbohydrate, protein, fat, fiber, SFA, PUFA, MUFA, cholesterol, vitamin E, vitamin B1, vitamin B2, niacin, folate, Na, K, Ca, P, iron, zinc, omega-3 fatty acid (*p* < 0.01). In addition, a negative relationship was observed between Mg intake and HIT-6 score (*p* < 0.05). Again, a negative relationship was observed between PSS-10 score and Mg intake (*p* < 0.05). According to the correlation between PSS-10 and HIT-6 scores, a positive relationship was observed (*p* < 0.01). A negative relationship was observed between the HIT-6 score and height, body weight, protein, cholesterol, vitamin B2, niacin, K and *p* values (*p* < 0.01).

**Table 6:** Correlation between age, dietary magnesium intake, height, body weight, BMI values, HIT-6, PSS-10 scores and nutrients.

Variables	Magnesium	Age	Height	Weight	BMI	HIT-6 score	PSS-10 score
Magnesium	1	0.156	0.257**	0.242**	0.124	−0.183*	−0.197*
Age	0.156	1	0.305**	0.311**	0.177*	−0.167*	−0.193*
Height	0.257**	0.305**	1	0.831**	0.272**	−0.313**	−0.338**
Weight	0.242**	0.311**	0.831**	1	0.758**	−0.271**	−0.331**
BMI	0.124	0.177*	0.272**	0.758**	1	−0.099	−0.172*
HIT-6 score	−0.183*	−0.167*	−0.313**	−0.271**	−0.099	1	0.456**
PSS-10 score	−0.197*	−0.193*	−0.338**	−0.331**	−0.172*	0.456**	1
Energy	0.685**	0.138	0.369**	0.328**	0.136	−0.209*	−0.208*
Carbohydrate (g)	0.594**	0.128	0.328**	0.274**	0.094	−0.186*	−0.160
Carbohydrate (%)	−0.076	−0.027	−0.012	−0.054	−0.069	−0.025	0.052
Protein (g)	0.612**	0.135	0.346**	0.316**	0.139	–0.262**	−0.206*
Protein (%)	0.020	0.018	0.041	0.057	0.045	−0.099	−0.038
Fat (g)	0.637**	0.101	0.322**	0.297**	0.136	−0.155	−0.197*
Fat (%)	0.069	−0.008	−0.038	0.000	0.039	0.116	−0.007
Fiber	0.645**	0.167*	0.154	0.186*	0.137	−0.142	−0.206*
Alcohol	0.050	0.125	0.155	0.165*	0.101	−0.056	−0.127
SFA	0.499**	0.102	0.284**	0.240**	0.081	−0.100	−0.147
MUFA	0.612**	0.105	0.350**	0.361**	0.207*	−0.168*	−0.262**
PUFA	0.535**	−0.032	0.196*	0.171*	0.074	−0.138	−0.087
Cholesterol	0.281**	0.139	0.336**	0.342**	0.184*	−0.252**	−0.205*
vit A	0.023	0.076	0.220**	0.268**	0.188*	−0.109	−0.073
vit E	0.554**	0.076	0.193*	0.180*	0.089	−0.122	−0.180*
vit K	0.081	0.031	−0.137	−0.112	−0.044	0.031	−0.066
B1	0.725**	0.128	0.272**	0.260**	0.124	−0.208*	−0.208*
B2	0.427**	0.240**	0.346**	0.376**	0.229**	−0.272**	−0.231**
B12	0.076	0.129	0.296**	0.341**	0.226**	−0.171*	−0.121
Niacin	0.534**	0.051	0.283**	0.249**	0.099	−0.259**	−0.165*
Folate	0.417**	0.208*	0.179*	0.244**	0.199*	−0.186*	−0.183*
C vit	0.182*	0.101	−0.160	−0.097	0.014	−0.146	−0.172*
Sodium	0.369**	0.145	0.369**	0.342**	0.165*	−0.129	−0.154
Potasium	0.704**	0.180*	0.221**	0.213**	0.105	−0.271**	−0.248**
Calcium	0.439**	0.287**	0.233**	0.220**	0.100	−0.053	−0.176*
Phosphorus	0.680**	0.239**	0.386**	0.355**	0.157	−0.220**	−0.214**
Iron	0.571**	0.097	0.128	0.128	0.062	−0.147	−0.212**
Zinc	0.277**	0.032	−0.046	−0.036	−0.022	−0.038	−0.131
Omega-3	0.392**	−0.033	0.197*	0.186*	0.099	−0.146	−0.019

BMI: body mass index; HIT: headache impact test; PSS: perceived stress scale; SFA: saturated fatty acids; MUFA: monounsaturated fatty acids; PUFA: polyunsaturated fatty acids. *p<0.05 and **p<0.01.

A negative relationship was also observed between age, energy, carbohydrate, MUFA, vitamin B1, vitamin B12 and folate values (*p* < 0.05). A negative relationship was found between the PSS-10 score and height, body weight, MUFA, vitamin B2, P and iron values (*p* < 0.01). Again, a negative relationship was observed between this scale score and age, BMI, energy, protein, fat, fiber, cholesterol, vitamin E, vitamin B1, niacin, folate, vitamin C and Ca values (*p* < 0.05).

## 4 Discussion

Individuals were divided into HIT-6 classes according to their Mg intake levels and gender, no significant relationship was found between the groups with adequate Mg and inadequate Mg intake (*p* > 0.05). However we found that the HIT-6 score was significantly higher in individuals receiving inadequate Mg compared to the group receiving adequate Mg (*p* < 0.05). In a study conducted on women in Brazil (28), individuals were divided into two groups as those with and without migraine diagnosis. Headache Impact Test-6 was used to measure the degree of headache in individuals. In addition, plasma Mg, selenium, Ca, copper, zinc and iron levels of individuals were examined. In addition, the intake of these minerals was also examined based on the retrospective 24-h food consumption record. As a result of the examination, it was observed that plasma Mg (*p* < 0.0001), Ca (*p* < 0.0001), copper (*p* < 0.001) and zinc (*p* < 0.001) values of individuals with migraine were significantly lower. In addition, it was observed that Mg, copper and iron taken from the diet were significantly lower in individuals with migraine (*p* < 0.05). In a study in China (29) aiming to examine the relationship between daily Mg and Ca intake of individuals and severe headache or migraine, it was found that individuals with headache had significantly lower levels of Mg intake compared to individuals without headache. Additionally, it was observed that Mg and Ca, alone or in combination, were negatively associated with migraine in women, whereas Ca was negatively associated with migraine in men.

The HIT-6 scores of the individuals in the study were analyzed according to gender, it was concluded that there was a significant difference between men and women (*p* < 0.001). In addition, it was observed that the number of males was significantly higher than the number of females in the no effect or very little effect group (*p* = 0.001), and the number of females was significantly higher than the number of males in the severe impact group (*p* < 0.001). The fact that the prevalence of migraine, which is one of the headache disorders, is 3–4 times higher in women than in men supports the results of this study (30). Cluster headache, which is among the primary group of headache disorders, has been known to affect men more, but it has been observed that there has been a steady decrease in the male:female prevalence ratio over time. It was also observed that the duration of pain was longer and the distribution of pain sites was wider in women than in men. It has also been observed that the onset of cluster headache in women usually coincides with periods of significant changes in hormone levels (menarche, postpartum, menopause) (31). Allais and colleagues (32) suggested that women’s susceptibility to migraine may be influenced by various factors, including hormones, brain structure, genetic polymorphisms or mutations, life events, stress, and neuronal activity. The vasodilatory effects of female sex hormones, particularly estrogen and progesterone, are known to involve multiple cellular mechanisms depending on the type of hormone and the target tissue. The greater susceptibility of women to migraine compared with men has been associated with the role of estrogens in modulating neuroexcitability in specific brain regions (33).

When the average of the PSS-10 scores of the individuals according to gender was taken, it was observed that the scores of the female individuals were significantly higher than the male individuals (*p* < 0.001). Again, according to the PSS-10 class distribution, it was seen that in the low-level stress class, the males were significantly more than the females (*p* < 0.05), and in the moderate-level stress group, the females were significantly more than the males (*p* < 0.001). In a study conducted on university students in the United States (34), when the stress levels of individuals were analyzed according to gender, it was seen that women’s stress levels were higher than men. It was observed that women were significantly more than men, especially in the moderate-level stress group (*p* < 0.001). A study involving licensed shooters affiliated with the Turkish Shooting and Hunting Federation across different competitive levels (elite, intermediate, and novice), female athletes were significantly higher PSS scores compared to male athletes (35) (*p* < 0.05).

In the study, when individuals were divided into PSS-10 classes according to Mg intake levels and gender, no significant relationship was found between the groups with adequate and inadequate Mg intake (*p* > 0.05). Since there were 15 individuals in the group with adequate Mg intake and 135 individuals in the group with inadequate Mg intake, it is expected that there would be no significant relationship. When the PSS-10 scores of the individuals were analyzed according to Mg intake and gender, it was concluded that although the stress level in the group with inadequate Mg intake was higher than in the group with adequate Mg intake, this difference was not significant (*p* > 0.05). However, we found a negative correlation between dietary Mg intake and PSS-10 score (*r* = −0.197, *p* < 0.05). It has been suggested in the literature that there is a negative relationship between Mg level in the body and stress (21). In addition, in a study conducted on university students in Japan (36) during a 4-week exam period, it was observed that all students experienced chronic sleep deprivation for 4 weeks, sleep lasted less than 80% of normal days, they were under great stress to pass the exams, and chronic stress significantly decreased erythrocyte Mg concentrations (from 5.7 ± 0.4 mg/mL to 5.5 ± 0.4 mg/mL, *p* < 0.05).

In this study, a negative relationship was found between dietary Mg intake and HIT-6 scores (*r* = −0.183, *p* < 0.05). In a study, it was observed that there was no significant relationship between the degree of headache effect and serum plasma and dietary Mg intake (*p* > 0.05) (28). In a study aiming to measure serum vitamin D, Ca and Mg levels in individuals with migraine, it was found that serum vitamin D and Mg concentrations of individuals with migraine were significantly low (*p* < 0.001 and *p* < 0.05, respectively). At the same time, vitamin D and Mg showed a negative significant relationship with migraine frequency, duration, severity and disability (37). It is known that Mg plays an important role in vitamin D synthesis and metabolism and that intestinal Mg absorption is vitamin D-dependent. Therefore, low vitamin D levels have been associated with low Mg levels (38).

In this study, a positive correlation was observed between PSS-10 and HIT-6 scores (*r* = 0.456, *p* < 0.01). In a study aiming to examine the prevalence and clinical characteristics of TTH and the psychosocial factors causing its onset and aggravation in university students in Türkiye, the prevalence of TTH was 20.35% (25.54% for women and 14.25% for men). Among headache sufferers, 43.7% were affected by one or more stressful life factors before the onset of headache and stress was recognized as the most common exacerbating factor (52%) (39). In a study of 418 individuals in Saudi Arabia (40), it was observed that only 8.9% of the participants met the ICHD-3 criteria for migraine headache screening and the majority were women (78.4%). The study showed a high prevalence of depression, anxiety and stress in the population (63.9, 63.6 and 55% respectively), with a higher prevalence in women. It was also observed that depression, anxiety and stress had a prevalence of 78.4% in individuals with migraine, which was significantly higher than in those without migraine.

In this study, the primary aim was to examine both the relationship between stress level and headache and the relationship of dietary Mg on these two factors in academic and administrative staff, which are professions with high workload. Our data showed that dietary Mg intake was inversely associated with HIT-6 and PSS-10. In addition, the relationship between HIT-6 and PSS-10 score is directly proportional. There is no study in the literature examining the relationship between dietary Mg intake and stress level and headache among academicians and administrative staff. Although there are no studies that have simultaneously addressed dietary Mg intake, stress levels, and headaches, most studies have reported an association between stress and headache. The results of our study are consistent with those previously reported in the literature. However, numerous underlying factors contributing to stress and headache. Dietary Mg is intake may be one of these factors.

The limitation of our study is that, we examined only the Mg intake derived from individuals’ diets. Since these values are subjective, possible inaccuracies should be taken into account. In future studies, including serum or erythrocyte Mg levels alongside dietary Mg will reduce the margin of error. A limitation of this study is the imbalance in sample sizes between the groups, with significantly fewer participants in the adequate Mg intake group (*n* = 15) compared to the inadequate intake group (*n* = 135). The small size of the adequate Mg intake group makes the results more susceptible to the influence of individual outliers, which may affect the overall generalizability of the findings. Additionally, the generalizability of our findings is limited due to specific inclusion criteria in our study. We limited our study sample to academic and administrative staff. By focusing exclusively on normal-weight academic and administrative staff from universities in Istanbul, the results may not be applicable to populations with different BMI profiles, such as individuals categorized as underweight, overweight, or obese. The sample group can be expanded (e.g., academic, administrative staff, university students etc.) and the effects of Mg, stress levels and headaches between groups can be examined. For instance, given that future-related anxiety is more prevalent among students, the outcomes may differ considerably within this group. Conducting such a study in the general population may allow for more comprehensive interpretations. Additionally, stress levels across occupations, degree of headache, dietary Mg intake, and serum or erythrocyte Mg levels in the general population can also be compared. We limited our work to Istanbul. The verifiability of the study will be strengthened by increasing the number of cities where the study is conducted.

## 5 Conclusion

Consequently, our findings indicate that dietary magnesium intake may be related to stress levels and headache outcomes among academic and administrative staff. Although the observed correlations were weak, lower dietary Mg intake was moderately associated with higher HIT-6 and PSS-10 scores. Adequate dietary Mg intake may be associated with lower levels of stress and headache.

## Data Availability

The datasets presented in this study can be found in online repositories. The names of the repository/repositories and accession number(s) can be found in the article/supplementary material.
